# The complete mitochondrial genome of the freshwater crab *Bottapotamon lingchuanense* Türkay and Dai 1997 (Decapoda: Brachyura: Potamoidea)

**DOI:** 10.1080/23802359.2021.1915208

**Published:** 2021-04-26

**Authors:** Yi-fan Wang, Qian Yang, Shu-xin Xu, Meng-jun Zhao, Jie-xin Zou

**Affiliations:** a School of Basic Medical Sciences, Research lab of Freshwater Crustacean Decapoda & Paragonimus, Nanchang University, Nanchang, PR China; b Institute of Pathogen Biology, Jiangxi Academy of Medical Sciences, Nanchang, PR China; c Department of Parasitology, School of Basic Medical Sciences, Nanchang University, Nanchang, PR China

**Keywords:** Brachyuran, mitochondrial genome, *Bottapotamon lingchuanense*, phylogenetics

## Abstract

We report the complete mitochondrial genome of *Bottapotamon lingchuanense* for the first time, which is found to be 17,612 base pairs in length, and contains 13 protein-coding genes (PCGs), 2 ribosomal RNA (rRNA) genes, 22 transfer RNA (tRNA), and 1 non-coding AT-rich region known as the D-loop. In addition, the mitogenome has 17 intergenic regions ranging from 1 to 1512 bp in length. The mitochondrial genome of *B. lingchuanense* is the first mitochondrial genome under the genus *Bottapotamon*, providing DNA data for species identification, enriching the species diversity of Brachyura. The maximum-likelihood (ML) tree and Bayesian inference (BI) tree based on the 13 PCGs of mitochondrial genome of Brachyura species showed similar topologies with high confidence, and the analysis results were consistent with the current mainstream classification system. The results indicating that *B. lingchuanense* is closely related to *Neilupotamon sinense*, *Sinopotamon*, and *Tenuilapotamon*, and it is likely to be derived from them.

The *Bottapotamon* is a genus of freshwater crab that is unique to mainland China. Up to now, there are eight species under the genus (Bott 1967; Dai 1999; Gao et al. 2019). In mainland China, the species under the *Bottapotamon* are mainly distributed within the area of the Wuyi Mountain Range, and only the species of *Bottapotamon lingchuanense* have been isolated in the Nanling Mountain Range (Türkay and Dai 1997). The terrain of the habitat of *B. lingchuanense* is geologically stable, as there is hardly affected by the Cenozoic orogeny (Gao et al. 2019). Therefore, we believe that the distribution of *B. lingchuanense* in mainland China is caused by the emergence of the mountains. At present, there is no mitochondrial genome data of the species under the genus *Bottapotamon* in GenBank. Here we present the first mitogenome for the genus *Bottapotamon* by sequencing the mitochondrial genome of the freshwater crab *B. lingchuanense*, which has a new mitogenome protein-coding gene order for the Decapoda, aiming to explore the evolutionary relationship within the genus *Bottapotamon*.

A male adult specimen of *B. lingchuanense* was collected from Yuanpu Village, Guanyin Township, Gongcheng County, Guilin City, Guangxi Province, China (**latitude** 110.9598 and **longitude**25.1634) in 2017. The sample is *B. lingchuanense*, which was identified by morphological methods. The specimen was deposited at the Laboratory Specimen Library of Freshwater Crustacean Decapoda & Paragonimus, School of Basic Medical Sciences, Nanchang University, Nanchang, Jiangxi, PR China & National Parasite Germplasm Resources Specimen Library of China (Jie-xin Zou jxzou@ncu.edu.cn) under the voucher number NCUMCP4076. The sample was fixed in ethanol and then stored at −20 °C before sequence analyses. Genomic DNA extraction, sequencing, gene annotation was performed referred to the ways routinely used before (Plazzi et al. 2013).

The complete mitogenome of *B. lingchuanense* is 17,612 bp in length (GenBank accession number: MN117717) with high A + T bias (72.33%), containing 13 protein-coding genes (PCGs), 2 ribosomal RNA (rRNA) genes, 22 transfer RNA (tRNA), and a non-coding AT-rich region and known as the control region. Not as usual, in *B. lingchuanense*, we can only clearly annotate 20 of the 22 tRNA genes, the positions, and structures of tRNA-Ile and tRNA-Met are slightly abnormal. By aligning it with genetic sequences of near-source species, we can basically confirm that the positions of tRNA-Ile and tRNA-Met should be located in the intergenic region of 149 bp between 12 S rRNA and ND2, and the first 66 bp area is determined as a special tRNA-Ile whose anti-codon is also converted from the usual GAT to AAT. However, the tRNAscan-SE software could not predict the secondary structure of the tRNA, and it could not be spliced into a regular shape by artificial folding neither. The post-83 bp area is the position of the putative tRNA-Met. Compared with the general tRNA-Met (about 70 bp), it is obvious that the length of tRNA-Met in *B. lingchuanense* is markedly longer. It is suspected that the deletion or replication of the base caused the abnormal of the tRNA genes, which may be caused by the mtDNA copy base deletion mutation.

Using the 48 Brachyura species published by GenBank and the 13 PCGs of mitochondrial genome of *B. lingchuanense* obtained in this study for phylogenetic analysis, *Kiwa tyleri* was selected as the outgroup, building maximum-likelihood (ML) and Bayesian inference (BI) trees, respectively. The ML tree and the BI tree obtained by the phylogenetic analysis showed similar topological structure and supported by a high degree of confidence (Figure 1). The results of phylogenetic analysis showed that, consistent with the results of morphological classification analysis, *B. lingchuanense* with unique gene arrangement and *Neilupotamon sinense* were clustered together, and they were combined with *Sinopotamon* and *Tenuilapotamon*. The composed clades form sister clades, indicating that *B. lingchuanense* is closely related to *Neilupotamon sinense*, *Sinopotamon*, and *Tenuilapotamon*, and it is likely to be derived from them.

**Figure 1.**
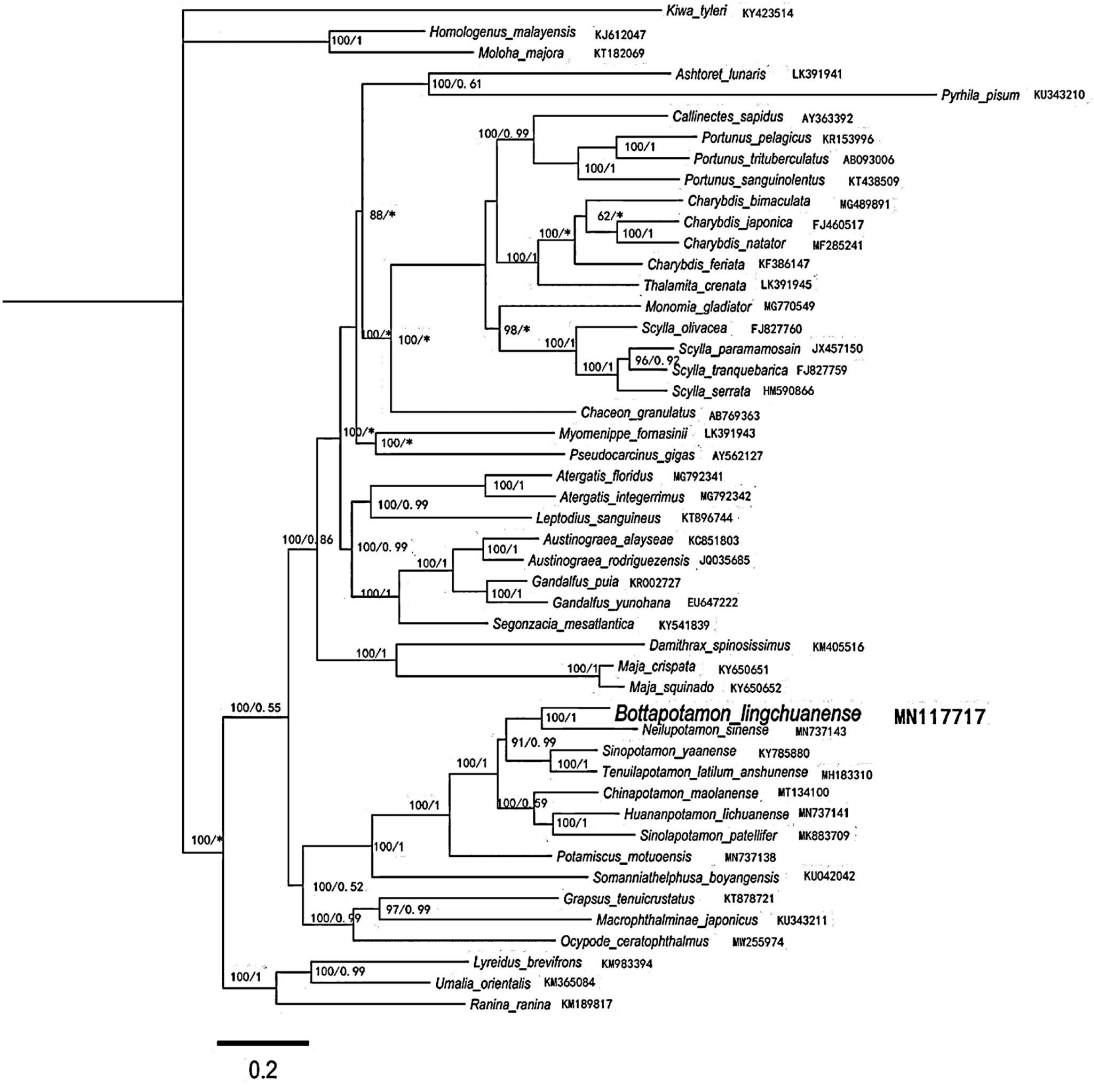
Phylogenetic maximum-likelihood (ML) tree of *Bottapotamon lingchuanense* and related brachyurans based on 13 PCGs nucleotide sequences from the mitochondrial genome. The numbers are Bayesian inference (BI) proportions and ML proportions. The differences between the ML and BI trees are indicated by ‘*’. The scale bars represent distance.

## Data Availability

The genome sequence data that support the findings of this study are openly available in GenBank of NCBI at https://www.ncbi.nlm.nih.gov/ under the accession no. MN117717.
